# Role of membrane porosity in passive sampling of aquatic contaminants for stable isotope analysis: enhancement of analyte accumulation rates and selectivity

**DOI:** 10.1007/s00216-025-05756-9

**Published:** 2025-01-31

**Authors:** Armela Tafa, Anat Bernstein, Martin Elsner, Rani Bakkour

**Affiliations:** 1 TUM School of Natural Sciences, Chair of Analytical Chemistry and Water Chemistry, Technical University of Munich, Lichtenbergstraße 4, 85748 Garching, Germany; 2 The Zuckerberg Institute for Water Research, The Jacob Blaustein Institutes for Desert Research, Ben-Gurion University of the Negev, Sde Boker Campus, 84990 Beersheba, Israel

**Keywords:** Passive sampling, Membrane porosity, Compound-specific isotope analysis, Selectivity, Biofouling, Aquatic contaminants

## Abstract

**Abstract:**

Compound-specific isotope analysis (CSIA) is a potent method for illustrating the in situ degradation of aquatic contaminants. However, its application to surface and groundwater is hindered by low contaminant concentrations, typically in the nanogram-per-litre range, requiring the processing of large water volumes. Polar organic chemical integrative samplers (POCIS) have shown promising results when combined with CSIA, yet their extended deployment time to accumulate sufficient analyte mass remains a major limitation. In our study, we addressed this issue by increasing the pore size of the polyethersulfone membrane (PES) from 0.1 to 8 $\upmu$m. This resulted in significant increases in the mass accumulation rates of atrazine (3.5-fold), S-metolachlor (3.4-fold), and boscalid (3.0-fold). Importantly, the larger pore sizes did not compromise isotopic integrity, with $\Delta \delta ^{13}$C$\le +0.4\pm 0.1$‰ and $\Delta \delta ^{15}$N$\le -0.6\pm 0.4$‰, both within accepted uncertainties. Additionally, we observed an enhanced selectivity of the larger pores towards the target analytes over humic acids, whereas no significant increase in (bio)fouling potential was detected for the 8 $\upmu$m membrane, as demonstrated by gravimetric analysis, SEM measurements, mass accumulation rates, and isotope ratios of fouled and unfouled POCIS. Our findings show that increasing the membrane pore size from 0.1 to 8 $\upmu$m reduces deployment time and expedites the accumulation of analyte mass required for gas chromatography isotope ratio mass spectrometry, offering a promising method to expand CSIA for low-concentration pesticide analysis in the field.

**Graphical abstract:**

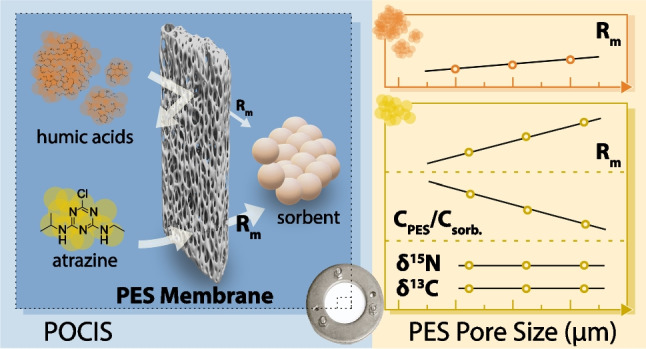

**Supplementary Information:**

The online version contains supplementary material available at 10.1007/s00216-025-05756-9.

## Introduction

Frequent detection of agricultural pesticides in streams and groundwater is a significant global concern, as it contributes to non-point source pollution and threatens ecological systems and human health [1–5]. For instance, the herbicide atrazine and its degradation products are still omnipresent in European waters today, with concentrations ranging from a few ng$\cdot$L$^{-1}$ up to low $\upmu$g$\cdot$L$^{-1}$, despite the EU’s ban in 2004 [6–8]. This poses challenges for societies in ensuring the safe consumption of water and the production of food [9, 10]. Therefore, understanding natural processes that may lead to pesticide degradation, such as biotic and abiotic transformation, in contaminated sites is crucial for effective environmental risk assessment. Screening and monitoring pesticide concentrations over time are, therefore, very valuable for assessing contaminated sites. However, they provide only limited insights into natural processes that may lead to (bio)degradation, as establishing mass balances and detecting metabolites can be challenging [11]. In contrast, compound-specific isotope analysis (CSIA) has been established in the last two decades as a powerful complementary method for evaluating the origin and fate of organic contaminants in environmental systems, where the ratio of stable isotopes (e.g., $^{13}$C/$^{12}$C, $^{2}$H/$^{1}$H, and $^{15}$N/$^{14}$N) can distinguish between sources of chemically identical substances and help decipher transformation mechanisms [12–15]. While significant changes in isotope ratios of the remaining unreacted pesticide provide conclusive evidence of (bio)transformation [16], this evidence is most visible in compartments where (bio)transformation has proceeded the furthest, resulting consequently in lower concentrations of the unreacted pesticide [17]. These low concentrations render the application of CSIA in the field analytically challenging [18, 19]. For instance, to accurately measure carbon isotope ratios using gas chromatography-combustion-isotope ratio mass spectrometry (GC-c-IRMS), a minimum of 1–10 nmol of carbon on the column is required [20]. This necessitates extraction of tens to hundreds of liters of water when pesticide occurrence is in the low ng$\cdot$L$^{-1}$ range, rendering the analytical process extremely laborious.

Passive samplers offer promise for field CSIA, serving not only as a sampler device, but also as an in situ pre-concentration tool, providing time-weighted average (TWA) concentrations of analyte over an extended deployment time, ranging typically from 30 to 60 days [21–23]. While passive samplers are widely used for screening purposes, only a few types have been tested for compatibility with CSIA and have been proven to preserve the isotopic signature of the sampled compounds. For instance, Passeport et al. [24] used passive equilibrium dialysis samplers, known as peepers, and reported that they preserved the isotopic signatures of chlorinated aromatic compounds, while Wang et al. [25] demonstrated the use of semi-permeable membrane devices (SPMDs), with insignificant C and H isotope fractionation for polycyclic aromatic hydrocarbons. In contrast, Goli et al. [26] reported a reproducible isotope shift ($\Delta \delta ^{13}$C = 0.4–1.9‰) for Waterloo membrane passive sampler. In particular, the polar organic chemical integrative sampler (POCIS), which consists of OasisHLB sorbent encased between two polyethersulfone (PES) membranes ($0.1{\upmu }$m) [23, 27–35], has shown promising results when combined with CSIA for a number of compounds. While Gilevska et al. [22] reported no isotopic shifts for common pesticides, Suchana et al. [21] and Vinyes-Nadal et al. [36] observed slight isotopic shifts for substituted chlorobenzenes and methoxychlor, respectively. Although POCIS has been shown to preserve the isotopic signatures of a number of analytes, the extended deployment time required to accumulate sufficient mass for CSIA remains a bottleneck. This motivates the need for modifications to samplers to enhance analyte accumulation in the sorbent and reduce deployment time for the following reasons:

(i) Deployment time decreases as the target analyte concentration increases, which implies that degraded pesticides with low concentrations require significantly longer sampling durations.
(ii) The potential of biofouling increases with extended de-ployment [37, 38], which may lead to biotransformation of the target analyte during sampling [21], resulting in an isotopic shift as well as clogging of the membrane pores and diminishing of sampling rates [39, 40].
(iii) Lastly, co-extraction of natural organic matter present in water would conceivably increase with deployment times requiring more extensive clean-up of the resulted extracts.

In fact, one of the major roles of the PES membrane (0.1 $\upmu$m) in a POCIS sampler is to dampen the fluctuations in its uptake kinetics as a result of changing flow rates in the surrounding water in order to simplify its calibration—converting the accumulated mass on the sorbent to TWA concentration in the investigated waters [27, 31–35, 41, 42]. Note that the latter is not a concern for isotope analysis, as long as these fluctuations do not induce an isotopic shift. Few studies have actually suggested that increasing the membrane pore size can favor the analyte transfer from the water to the sorbent [43]. Belles et al. [44] reported a significant increase in sampling rates of different analytes when enlarging the membrane pore size to 30 $\upmu$m using nylon membranes, while MacKeown et al. [35] observed a similar effect with PES membranes at a pore size of 5 $\upmu$m, compared to the conventional 0.1 $\upmu$m membranes. As a matter of fact, enlargement of the membrane pore size seems to be ideal to enhance the mass accumulation rate of organic analytes (*R*_m_), obtaining thereby the critical mass required for CSIA in a shorter deployment duration.

Therefore, the main objective of this study was to enhance the sensitivity and selectivity of POCIS packed with OasisHLB to suit CSIA by enlarging the PES membrane pore sizes from the conventional design (PES 0.1 $\upmu$m) to 5 and 8 $\upmu$m of the same thickness. Three common pesticides, namely, atrazine (ATZ), S-metolachlor (MET), and boscalid (BOS), were used as model target analytes. Our specific objectives were (i) to measure mass accumulation rates (*R*_m_) and C_PES_/C_HLB_ of the investigated analytes, (ii) to evaluate the integrity of their $\delta ^{13}$C and $\delta ^{15}$N values, (iii) to quantify the mass accumulation rates of typical aquatic interferences for isotope analysis, such as humic acid, and lastly (iv) to assess the potential of (bio)fouling of POCIS through measurement of mass accumulation rates of ATZ, its $\delta ^{13}$C, gravimetric analysis and scanning electron microscopy of the different membranes and/or sorbent.

## Experimental section

### Chemicals and materials

The supporting information provides a list of acquired chemicals and materials (Table S1) and a detailed description of the preparation of standard solutions and working solutions used in this research (S1).

### Preparation, deployment, and extraction of POCIS

Construction of the in-house POCIS involved assembling a membrane–sorbent–membrane sandwich configuration after Alvarez et al. [23].

#### Pre-purification of membranes and sorbents

Prior to POCIS construction, purification procedures for the PES membranes and the OasisHLB sorbent were performed. To this end, 10 PES membranes were placed in a glass beaker containing 40 mL of methanol/water (20/80 v/v), then covered and left to soak overnight. This process was repeated twice after replacing the solvent mixture with methanol (100%) followed by a drying step using a gentle nitrogen stream. The purified dry PES membranes were then wrapped in aluminum foil, which has undergone the same purification process, and stored in a freezer at −20 °C until further use. As for OasisHLB, 10–15 g were loosely packed in a glass column typically used for flash chromatography and subjected to a sequential washing with methanol, methyl tertiary-butyl ether, dichloromethane, and methanol using an SPE vacuum manifold (Visiprep, Sigma-Aldrich, Germany). The volume of each solvent was 250 mL, and the flow rate was maintained constant at roughly 1.5 mL$\cdot$min$^{-1}$. The sorbent was exposed to flowing air using a vacuum pump until dryness and then transferred to pre-cleaned glass bottles until further use.

#### Assembly and deployment of POCIS

Initially, one PES membrane was placed at the bottom of a compression holder washer and the Oasis HLB sorbent (200 ± 5 mg) was carefully arranged in the center of the membrane, which was then covered with the second PES membrane. A second compression holder washer was placed at the top and secured with wing nuts to prevent any loss of the sorbent. In addition to the conventional POCIS configuration with 0.1 $\upmu$m membrane, we prepared two modified POCISs with pore sizes of 5 and 8 $\upmu$m that underwent the same preparation procedure. Deployment of the assembled POCISs was done in glass beakers for all experiments with the three configurations of POCIS (i.e., PES membranes 0.1, 5 and 8 $\upmu$m), where no renewal of water was performed unless mentioned otherwise. The deployment conditions were as the following: (a) For determination of mass accumulation rates of the investigated pesticides, three passive samplers of each configuration were deployed in 1 L of deionized water, and the deployment beaker was spiked with a mixture of ATZ, MET, and BOS to reach a concentration of 120 $\upmu$g$\cdot$L$^{-1}$ for each pesticide. The pot was then stirred using a hot-plate stirrer (1500 rpm) and a magnetic bar (*d* = 4.2 cm) at a constant speed for 6 days. (b) For determination of accumulation rates of NOM and ATZ, two experiments were conducted in parallel. One beaker was filled with deionized water and spiked with 4.0 mg$\cdot$L$^{-1}$ of HA, whereas the second beaker was spiked with 20 $\upmu$g$\cdot$L$^{-1}$ of ATZ. Deployment of POCISs was performed in each beaker for 7, 14, and 21 days. (c) Experiments for investigating the impact of (bio)fouling on POCIS were performed after Rosen et al. [38] entailing a pre-fouling step achieved through deploying the assembled POCIS configuration in river water collected from the Altmühlmünster river, near Riedenburg in Germany (48°58$'$58.6$''$N, 11°37$'$15.2$''$E) for 21 days. On day 21, water was changed from river to deionized water with a total volume of 15 L and spiked with ATZ at a concentration of 2 $\upmu$g$\cdot$L$^{-1}$ and stirred for another 9 days. Control experiments were conducted in parallel in a separate beaker under identical conditions and for 9 days, but without the pre-fouling step in river water.

During deployment, water flow was simulated in the corresponding beaker using an overhead stirrer equipped with an impeller (Heidolph Hei-Torque, Germany) that was set to a value corresponding to a linear velocity ($V=$ 0.3 m$\cdot$s$^{-1}$) according to Eq. 1, whereas a temperature of 25 °C was maintained during the deployment duration.

1 $$\begin{aligned} V = \frac{D^{2}\cdot \rho \cdot \upsilon }{\alpha \cdot \mu }\times \frac{RPM}{60} \end{aligned}$$

where *D* is the length of the impeller (given in m); $\rho$, $\upsilon$, and $\mu$ are the density (g$\cdot$cm$^{-3}$), kinematic viscosity (m$^{2}\cdot$s$^{-1}$), and dynamic viscosity of water (g$\cdot$cm$^{-1}\cdot$s$^{-1}$), respectively, at 25 °C; $\alpha$ is the diameter of the deployment beaker (m); *RPM* is the number of revolutions of the propeller per time (min$^{-1}$); whereas 60 is a conversion factor (s$\cdot$min$^{-1}$).

#### Retrieval and extraction after deployment

Retrieval of the deployed POCISs was performed at the end of the corresponding deployment duration, where each POCIS was disassembled. The semi-wet sorbent was carefully transferred, with the help of several MilliQ water washes, to a pre-weighted SPE cartridge equipped with a Teflon fret and left to dry at room temperature overnight. The recovered sorbent mass was quantified gravimetrically, and further processing was only continued if the recovery was $\ge$ 98%. In parallel, the two disassembled PES membranes were soaked in MilliQ water for 5 min, then washed with another 10 mL of MilliQ water, to remove any sorbent residuals from the experiments, followed by 4 h of drying at room temperature. Elution of the sorbates on the sorbent was carried out by percolating 10 mL of methanol into the prepared SPE cartridge, and the eluate was divided into two parts from which a 200 $\upmu$L was used for the analyte quantification, whereas the remaining volume was further reduced using an evaporation device (TurboVap® LV, Biotage, Sweden) for stable isotope analysis. The dried membranes were transferred into glass flasks, followed by extraction with 30 mL of methanol while stirring for 30 min at 450 rpm. An aliquot of 200 $\upmu$L was used for analyte quantification. The flask was weighed before and after stirring to account for any possible solvent evaporation. As for the DOC quantification, the 10 mL of sorbent extract and the 30 mL of membrane extracts were evaporated to dryness, reconstituted each with 10 mL of MilliQ water, and measured using a TOC analyzer.

### Chemical and isotope analyses

#### Pesticides quantification using GC-MS

ATZ, BOS, and MET were quantified using a gas chromatograph (GC 7890A) coupled to a quadrupole mass spectrometer (MS 5979C), both from Agilent Technologies. Prior to measurements, the samples were spiked with a mixture of internal standards. A 1 $\upmu$L volume was injected by a PAL autosampler (CTC, Switzerland) into a split/splitless injector equipped with a splitless liner (5 mm ID$\times$105 mm L, Thermo Fisher Scientific, Germany) at a temperature of 250 °C. Chromatographic separation of the analytes was achieved using an Rxi$^{\circledR }-$ 5ms column (Restek, USA) (30 mL $\times$ 0.25 mm ID $\times$ 0.25 $\upmu$m film thickness) operated with a programmed temperature gradient (see S2 for details). The helium carrier gas was maintained at a constant flow rate of 1.4 mL.min$^{-1}$. MS data were acquired in SIM mode using the assigned quantifier *m*/*z* for each analyte at its respective retention time (Table S2). The ion source and quadrupole temperatures were maintained at 230 and 150 °C, respectively. Pesticide concentrations in the injected extracts, $C_{i, inj}$, were calculated by comparing the peak area ratio of the quantifier ion and its assigned internal standard with the corresponding ratio in a calibration curve (see Table S2 for LODs and LOQs). The accumulated mass of each pesticide, $m_{i}$ in g, on 1 POCIS (= 2 PES membranes and 200 mg OasisHLB) was then calculated using Eq. 2:

2 $$\begin{aligned} m_{i} = C_{i, inj} \cdot V_{ext} \end{aligned}$$

where $C_{i, inj}$ is the calculated concentration of pesticide *i* in the injected sample of the corresponding extract (g$\cdot$L$^{-1}$); $V_{ext}$ is the volume of the extract (= 0.01 L for OasisHLB; = 0.03 L for PES).

**Fig. 1:**
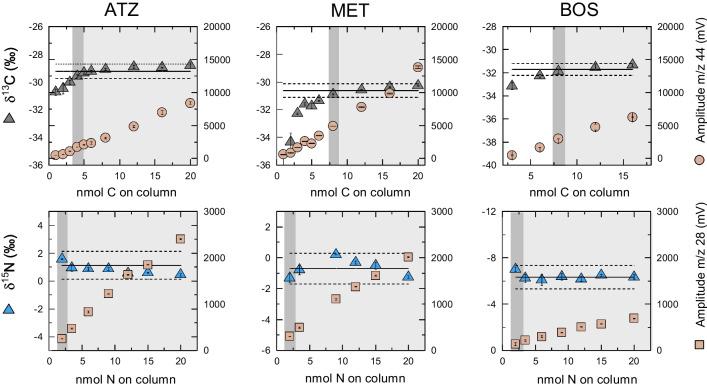
Carbon and nitrogen stable isotope ratios of atrazine (ATZ), boscalid (BOS), and S-metolachlor (MET) as a function of injected amount of C and N on column using GC-c-IRMS. Triangles represent the mean of triplicate measurements of $\delta ^{13}$C (black) and $\delta ^{15}$N (blue), both given in ‰, whereas orange circles and squares represent the signal amplitude of m/z 44 for C and m/z 28 for N, respectively, given in mV. Horizontal solid lines represent the true value of $\delta ^{13}$C and $\delta ^{15}$N. Gray vertical bars refer to the precise method quantification limit for each compound after Jochmann et al. [46] within the typical uncertainty of $\pm 0.5$ and $\pm 1.0$ ‰ for C and N, respectively, represented as horizontal dashed lines. Error bars reflect the standard deviation of triplicate measurements

#### Humic acid quantification using TOC analyzer

Humic acids (HA) in obtained extracts were quantified after evaporation and reconstitution in water as non-purgeable organic carbon using a total organic carbon analyzer (TOC-L, Shimadzu, Japan) equipped with a combustion catalytic oxidation reactor (680 °C) and a non-dispersive infrared (NDIR) detector. The instrument was calibrated before each sequence by measuring standard solutions of potassium hydrogen phthalate. Quality control measurements of blanks, constantly run alongside samples, ensured background DOC values were below the instrument’s detection limit (0.5 mgC$\cdot$L$^{-1}$). Accumulated mass of HAs on sorbent and membrane, $m_{HA}$ in g, was calculated according to Eq. 3:

3 $$\begin{aligned} m_{HA} = C_{HA} \cdot V_{rcst}/0.5 \end{aligned}$$

where $C_{HA}$ is the concentration of HA in the obtained extracts after reconstitution in water (gC$\cdot$L$^{-1}$); $V_{rcst}$ is the reconstituted volume of the corresponding extract in water prior to TOC measurement (= 0.01 L); and 0.5 represents the carbon contents of humic acid to convert from gC$\cdot$L$^{-1}$ to gHA$\cdot$L$^{-1}$.

**Table 1:** Method quantification limits (MQLs) of precise carbon and nitrogen stable isotope analysis determined according to the moving mean procedure after Jochmann et al. [46]

Analyte	MQL (nmol C)	Amplitude (m/z = 44)	MQL (nmol N)	Amplitude (m/z = 28)
ATZ	4	1728±21	2	253±1
MET	8	4939±4	2	303±3
BOS	8	2294±25	2	140±17

MQLs are expressed as injected mass on column in nmol C and nmol N, with corresponding peak amplitudes at m/z 44 and 28 mV, respectively

#### Carbon and nitrogen isotope ratio measurements on GC-c-IRMS

Isotope ratio measurements of ATZ, MET, and BOS were conducted on a GC-c-IRMS system, which consisted of a TRACE GC Ultra gas chromatograph coupled with a Finnigan MAT 253 isotope ratio mass spectrometer via a Finnigan GC Combustion III interface (Thermo Fisher Scientific, Germany). The method used in this work for chromatographic separation and data acquisition is described in detail by Glöckler et al. [45]. Briefly, a 3 $\upmu$L sample volume was injected in split/splitless mode at a temperature of 250 °C, where the injector was operated for 1 min in splitless mode with a pressure of 250 kPa, then switched to split mode with a flow rate of 20 mL$\cdot$min$^{-1}$. Analytes were separated on a temperature-ramped GC column (J&W DB-5MS UI column; 30 m L $\times$ 0.25 mm ID $\times$ 1.0 $\upmu$m film thickness, Agilent) protected by a 1-m guard column. Target analytes were combusted at 1050 °C in a custom-made oxidation reactor (Ni/Ni/Pt), and re-oxidized after each measurement for 20 min with O_2_, whereas nitrogen oxides were converted to N$_{2}$ at 650 °C in a subsequent reduction reactor (Cu). Isotope ratios were calibrated against a laboratory monitoring gas (CO$_{2}$ and N$_{2}$), itself calibrated against international reference standards USGS-61, USGS-62, and USGS-63 for carbon, and air, IAEA-600,USGS-62, and USGC-63 for nitrogen. Method quantification limits were determined using the moving mean method after Jochmann et al. [46] (see Fig. 1 and Table 1). Peak detection was performed by the software Iodate (Thermo Fisher Scientific, Germany), using the individual background algorithm for baseline correction. $\delta ^{13}$C and $\delta ^{15}$N are reported as arithmetic means of at least triplicate measurements given in ‰ relative to the international reference material Vienna Pee-Dee Belemnite (VDPB) and air, respectively.

### Assessment of (bio)fouling degree of POCIS using surface and gravimetric analysis

The potential of (bio)fouling on POCIS was assessed using pre-fouled POCIS as suggested by Rosen et al. [38] (see Section “Preparation, deployment, and extraction of POCIS”). After retrieval of the deployed POCISs on day 30, concentrations and carbon isotope values of ATZ were measured after extraction, to determine the analyte accumulation rate and validate the integrity of isotope analysis. Additionally, scanning electron microscope (SEM) measurements were performed on PES using a Sigma 300 VP instrument (Zeiss, Oberkochen, Germany) operating at an accelerated voltage of 10.0 kV. The acquired images were further processed using an open source software (ImageJ 1.54d, Java 1.8.0_345(64-bit)) developed by Wayne Rasband and contributors, National Institute of Health, USA [47]. The scale of the SEM images was first configured in the software to obtain the total area of the investigated image, $A_{SEM}$ given in $\upmu$m$^{2}$, followed by freehand selection of non-membrane materials, $A_{foreign}$ given in $\upmu$m$^{2}$, resulting in a surface coverage by foreign materials denoted as S$_{\text {fouling}}$ expressed in % according to Eq. 4.

4 $$\begin{aligned} \text {S}_{\text {fouling}} = \frac{A_{foreign}}{A_{SEM}} \times 100 \end{aligned}$$

The mass increase on fouled POCISs was assessed on both PES and OasisHLB by subtraction of their dry weight determined on an analytical balance before and after deployment and expressed as $\Delta$m$_{\text {fouling}}$ given in mg.

### Data evaluation

#### Mass accumulation rates of sorbates and their distribution ratios on POCIS phases

Sampling rates of sorbate *i*, namely ATZ, MET, BOS, and HA, are reported in this work as mass accumulation rates, $R_{m}$, on both phases of POCIS *j*, namely PES membrane or OasisHLB, and expressed in $\upmu$g$\cdot$d$^{-1}$ according to Eq. 5:

5 $$\begin{aligned} R_{\text {m}} = \frac{m_{\text {i},\text {j}}}{t} \times 1000 \end{aligned}$$

where *m* (given in g) represents the measured mass of sorbate *i* accumulated on the corresponding POCIS phase *j*; *t* is the deployment time (d).

Concentrations of sorbates *i* were calculated in both phases *j* to determine the ratio $C_{\text {i},\text {PES}}/C_{\text {i},\text {HLB}}$ according to Eq. 6:

6 $$\begin{aligned} \frac{C_{\text {i},\text {PES}}}{C_{\text {i},\text {HLB}}} = \frac{m_{\text {i},\text {PES}}/0.000572}{m_{\text {i},\text {HLB}}/0.0002} \end{aligned}$$

where $C_{\text {i},\text {PES}}$ is the concentration of *i* (= pesticide or HA), measured on the PES phase ($\upmu$g$\cdot$L$^{-1}$); $C_{\text {i},\text {HLB}}$ is the concentration of *i* on the OasisHLB phase ($\upmu$g$\cdot$kg$^{-1}$); 0.000572 is the volume of one PES membrane (L) based on its thickness of 110–150 $\upmu$m and exposure diameter of 53 mm; and 0.0002 is the packed mass of OasisHLB within the POCIS (kg).

Mass distribution of the sorbate *i* between the two phases, $M_{i,PES}$ and $M_{i,HLB}$, was calculated according to the Eqs. 7 and 8 and expressed in %.

7 $$\begin{aligned} M_{\text {i},\text {PES}} = \frac{m_{\text {i},\text {PES}}}{m_{\text {i},\text {HLB}}+m_{\text {i},\text {PES}}} \times 100\% \end{aligned}$$

8 $$\begin{aligned} M_{\text {i},\text {HLB}} = \frac{m_{\text {i},\text {HLB}}}{m_{\text {i},\text {HLB}}+m_{\text {i, PES}}} \times 100\% \end{aligned}$$

#### $\Delta \delta ^{13}$C and $\Delta \delta ^{15}$N

Shifts of isotopic signatures are reported in ‰as the deviation $\Delta \delta$ $^{13}$C and $\Delta \delta ^{15}$N between the measured isotope ratios in the processed sample ($\delta ^{13}$C$_{\text {Sample}}$ and $\delta ^{15}$N$_{\text {Sample}}$) and the same isotopic in-house standard ($\delta ^{13}$C$_{\text {Standard}}$ and $\delta ^{15}$N$_{\text {Standard}}$), as measured directly on GC-c-IRMS. Bracketed standards during measurements showed no isotopic shift throughout the sequence. Uncertainties associated with measurements were propagated according to the Gaussian error propagation law and are reported as 95% confidence intervals.

9 $$\begin{aligned} \Delta \delta ^{13} C= &   \delta ^{13}C_{\text {Sample}} - \delta ^{13}C_{\text {Standard}} \end{aligned}$$

10 $$\begin{aligned} \Delta \delta ^{15} N= &   \delta ^{15}N_{\text {Sample}} - \delta ^{15}N_{\text {Standard}} \end{aligned}$$

## Results and discussion

### Increasing membrane pore size enhances mass accumulation rates of ATZ, MET, and BOS on the sorbent and reduces the ratio $C_{\text {PES}}/C_{\text {HLB}}$

We tested the effect of increasing the porosity of PES membranes (i.e., 0.1, 5, and 8 $\mu$m) for POCISs immersed in water by assessing their corresponding mass accumulation rates for three selected pesticides (ATZ, BOS, and MET) at identical conditions of target concentration, using static calibration experiments (C=120 $\upmu$g$\cdot$L$^{-1}$), water velocity (*V*=0.3 m$\cdot$s$^{-1}$), and deployment time (*t*=6 days).

**Fig. 2:**
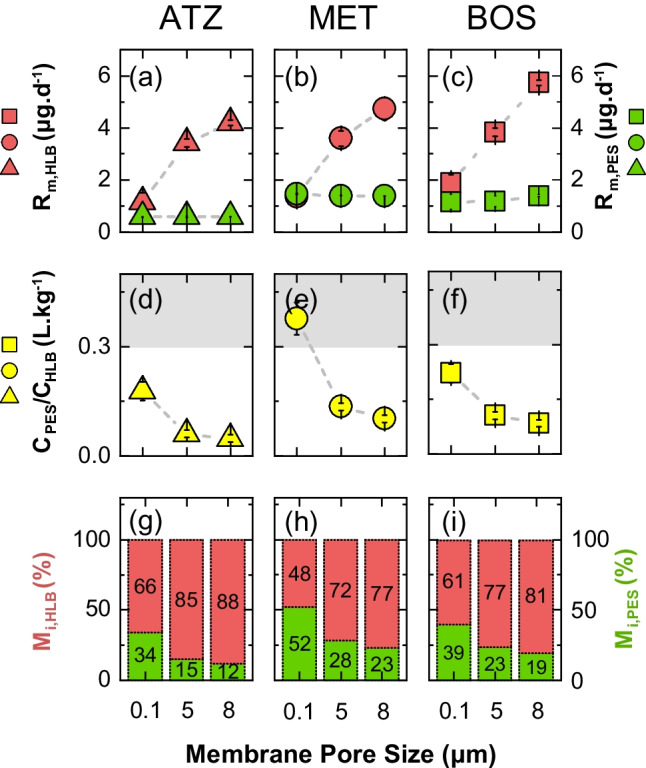
**a**–**c** The mass accumulation rates, R$_{\text {m}}$, of ATZ, MET, and BOS on the Oasis HLB sorbent (red symbols) and the PES membranes (green symbols) at different membrane pore sizes of 0.1, 5, and 8 $\upmu$m. **d**–**f** The ratio of the corresponding target concentrations between PES and HLB phases, C$_{\text {PES}}$/C$_{\text {HLB}}$ (yellow symbols), represents a proxy for mass transfer delay above the assigned region (grey area, C$_{\text {PES}}$/C$_{\text {HLB}}\ge$ 0.3 L$\cdot$kg$^{-1}$). **g**–**i** Mass distribution of the corresponding target analytes between the PES membrane (green columns, M$_{\text {i},\text {PES}}$) and the Oasis HLB sorbent (red column, M$_{\text {i},\text {HLB}}$). The dashed grey lines serve as visual guides, indicating the increasing or decreasing trends in relation to the membrane pore size for each compound

Figure 2 shows a progressive increase in the mass accumulation rates on OasisHLB phase (R$_{\text {m},\text {HLB}}$, red symbols in Fig. 2 a–c) for all three investigated compounds at large membrane pore sizes. These ranged from 1.2±0.3 $\upmu$g$\cdot$d$^{-1}$ (0.1 $\upmu$m) to 4.2±0.1 $\upmu$g$\cdot$d$^{-1}$ (8 $\mu$m) for ATZ, from 1.4±0.2 $\upmu$g$\cdot$d$^{-1}$ to 4.7±0.4 $\upmu$g$\cdot$d$^{-1}$ for MET, and from 1.9±0.2 $\upmu$g$\cdot$d$^{-1}$ to 5.7±0.5 $\upmu$g$\cdot$d$^{-1}$ for BOS, corresponding to a factor (8 $\upmu$m/0.1 $\upmu$m) of 3.5±0.2, 3.4±0.3, and 3.0±0.4, respectively. We attribute this significant gain in $R_{\text {m},\text {HLB}}$ to the substantial influence of the membrane pore size, which enhances the accumulation of the compounds onto the sorbent by reducing mass transfer resistance, improving their diffusive transport within the expanded pore network of the membrane. Conversely, mass accumulation rates on the PES phase ($R_{\text {m},\text {PES}}$, green symbols in Fig. 2 a–c) remain unaffected by the enlargement of the pore size.

This is further corroborated through the ratio of pesticide concentrations on both phases (= C$_{\text {PES}}$/C$_{\text {HLB}}$), where values > 0.3 imply as suggested by Vermeirssen et al. [43] a temporal delay as the compound diffuses from the aqueous boundary layer to the sorbent through the PES membrane pores. In fact, $C_{\text {PES}}$/$C_{\text {HLB}}$ for ATZ, MET, and BOS show a decreasing order with increasing membrane pore size (yellow symbols in Fig. 2 d–f). These changes in $C_{\text {PES}}$/$C_{\text {HLB}}$ correspond to a decrease of 50±5, 75±4, and 50±8% for ATZ, MET, BOS, respectively, when the 0.1 $\mu$m is replaced by the 8$\mu$m membrane. While these results are consistent with the faster diffusion through larger membrane pores, they also imply that the membrane with smaller pore size may act as a substantial sink. The mass distribution of the analytes between the two phases demonstrates that 34–52% of the analytes were retained by the 0.1 $\upmu$m PES membrane upon sampling, compared with 12–23% retained by the 8 $\upmu$m membrane (Fig. 2 g–i). As such, the necessity to extract both phases after POCIS sampling can be mitigated through the use of membranes with larger pores, thereby reducing the tediousness of multi-step extraction, combining extracts, and the associated risks of possible losses and biases.

The increase in $R_{\text {m},\text {HLB}}$ and the decrease in the ratio $C_{\text {PES}}$/$C_{\text {HLB}}$ promise good applicability for the investigated compounds to isotope analysis, as the critical mass can be obtained in a shorter deployment time. We presume that our findings are conceivably extendable to a larger set of compounds, considering previous studies on molecular interactions between PES membranes and aquatic organic contaminants. To this end, log $K_{\text {ow}}$ or log $D_{\text {ow}}$, as a proxy for the partitioning of compounds into the PES, has been proposed to give insights into mass accumulation rates [23, 36, 43, 48, 49]. For instance, Morin et al. [50] conclude that compounds with log D$_{\text {ow}}$ values between 2.3 and 3.6 do not accumulate within the PES membrane. Additionally, Harman et al. [49] noted the presence of a higher accumulation in the PES membranes, for compounds with log K$_{\text {ow}}>$ 3.1. Our findings are consistent with the ranges reported by Harman et al. [49] and Morin et al. [50], with log $K_{\text {ow}}$ values (ATZ = 2.6 [43], MET = 3.1 [51], and BOS = 3.0 [45]) fall in the lower end of these limits. In contrast, Vermeirssen et al. [43] concluded that after 32 days, the accumulated mass in the PES membrane becomes evident, for compounds with medium to higher log $K_{\text {ow}}>$ 2. We find our data most comparable to the findings of Harman et al. [49] and Morin et al. [50] since our compounds are within the linear range of the POCIS and have not reached equilibrium over the 6-day deployment time. Similarly, the partition equilibrium constant of analyte between the PES membrane and water, i.e., log $K_{\text {PES},\text {Water}}$, can also give insight into our presumption as a positive correlation between log $K_{\text {PES},\text {Water}}$ and log $K_{\text {ow}}$ has been reported [43, 51, 52], indicating that the compound affinity towards the membrane affects its diffusion through the latter and hence mass accumulation rates on the sorbent. Djomte et al. [51] concluded that compounds with log K$_{\text {PES},\text {Water}} \le$ 3.31 predominantly accumulate on the Oasis HLB sorbent, whereas those with log $K_{\text {PES},\text {Water}} \ge$ 3.91 mostly accumulate in the PES membranes. The results for ATZ (log K$_{\text {PES},\text {Water}}=3.25$ [43]; =3.31 [51]) and MET (log $K_{\text {PES},\text {Water}}=2.70$ [43]; = 3.05 [51]) align with these trends, as a high affinity for the sorbent is observed, whereas a value of log K$_{\text {PES},\text {Water}}$ for BOS could not be obtained.

**Fig. 3:**
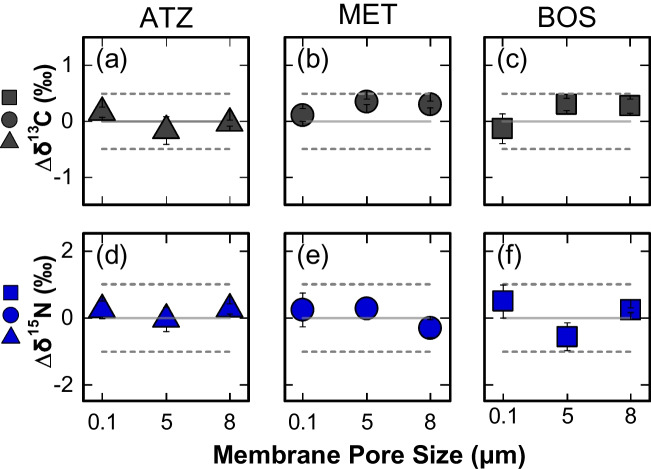
Stable isotope deviations from standard values for carbon (**a**–**c**) and nitrogen (**d**–**f**) measured for ATZ, MET, and BOS after sampling by POCIS packed with OasisHLB and equipped with PES membranes at porosities of 0.1, 5, and 8 $\upmu$m. The dashed lines represent the typically accepted uncertainties of ± 0.5‰ for $\delta ^{13}$C and ±1‰ for $\delta ^{15}$N

Based on these studies, one could anticipate a gain in accumulated mass, upon increasing the membrane porosity, for a neutral compound that has a similar or larger affinity towards the sorbent compared to the investigated compounds (log $K_{\text {d}} \ge$ 4.8 [45]) and a low affinity for the PES membrane (log $K_{\text {PES},\text {Water}} \le$ 3.3 [51]). In this context, Suchana and Passeport [52] and Dias and Poole [53] have provided invaluable works on the partitioning of compounds between water and different POCIS compartments (i.e., PES and Oasis HLB) using poly parameter linear free energy relationship (LFER) models. One could use these prediction tools along with the above-suggested values to predict the behavior of a compound during POCIS sampling. Examples of such compounds are alachlor, carbamazepine, and terbuthylazine (see Section S3, Table S3 for further compounds). Yet, it remains imperative to test the increase for each compound on a case-by-case basis by experimental measurement of their specific accumulated mass.

### No isotopic fractionation of $\delta ^{13}$C and $\delta ^{15}$N for ATZ, BOS, and MET

The primary aim of this study was to improve mass accumulation rates of pesticides during a reduced deployment time, facilitating the attainment of the critical mass necessary for GC-IRMS analysis. Figure 3 presents $\Delta \delta ^{13}$C and $\Delta \delta ^{15}$N values for ATZ, BOS, and MET measured in the extracts of the OasisHLB sorbent encased between PES membranes of different porosities (0.1, 5, and 8 $\upmu$m). The deployment conditions are identical to those used to assess R$_{\text {m}}$ (see Section “Increasing membrane pore size enhances mass accumula-tion rates of ATZ, MET, and BOS on the sorbent and reduces[Sec Sec17]”). The isotopic signatures of ATZ, BOS, and MET are preserved within the accepted uncertainties of ±0.5‰ for $\delta ^{13}$C and ±1‰ for $\delta ^{15}$N. This means that an accelerated diffusion process through the enlarged pores of the membrane followed by sorption on OasisHLB did not induce isotopic shifts.

Integrity of the $\delta ^{13}$C values of ATZ and MET for the 0.1 $\upmu$m pore size is consistent with findings of Gilevska et al. [22] with a 30-day deployment time. Note that no further isotopic data were found for BOS for any of the POCIS configurations. In contrast, Suchana et al. [21] reported slight isotopic fractionation on $\delta ^{13}$C and $\delta ^{15}$N values when studying the compatibility of conventional POCIS (sorbent = OasisHLB, membrane = PES 0.1 $\upmu$m) for four nitro- and amino-substituted chlorobenzenes. Suchana et al. [21] attributed this shift to the sorption of these compounds on the PES membrane, since no isotopic fractionation was observed during the conventional SPE using only Oasis HLB [54]. Similarly, Vinyes-Nadal et al. [36] reported a significant isotopic shift for methoxychlor extracted from the PES membrane ($\Delta \delta ^{13}$C = 6.3±0.1‰, log $K_{\text {ow}}$ = 5.08, log $K_{\text {PES},\text {Water}}$ = 6.17) upon investigation in a sorbent-free POCIS after 2 days of deployment.

Thus, the comparison suggests that combining POCIS with CSIA is a promising approach for compounds that do not predominantly accumulate in the PES membrane. Of course, the risk of observing an isotopic shift is not only the result of mass fractionation between the PES membrane and the sorbent, but it also depends on the magnitude of diffusion-/sorption-induced isotope effects on the PES. Therefore, it is not possible with our data set to define a mass fraction threshold beyond which no isotopic fractionation can be expected. Yet, increasing the PES membrane pore size led to a decreased mass fraction accumulating in the PES membrane, as demonstrated. This means that larger pores of the PES membrane not only decrease the time required to obtain the required mass for GC-IRMS, but also make the accumulation process less susceptible to isotope fractionation. Nonetheless, careful analytical validation of isotopic integrity is warranted for each compound and matrix before deployment in the field.

### Enhanced selectivity for ATZ due to exclusion of humic acids at larger membrane pore sizes

The increase in mass accumulation rates observed for pesticides in this work also implies that concurrent constituents in the water, such as natural organic matter (NOM), could potentially experience an enhanced accumulation in the sorbent at higher membrane porosities. Therefore, we investigated R$_{\text {m}}$ for NOM, as its presence may impact the isotopic signature, especially when the C$_{\text {DOM}}$/C$_{\text {analyte}}$ ratios are higher than 10 mol C$\cdot$mol C$^{-1}$ [45, 55, 56]. HAs were chosen as a representative model for NOM since it constitutes approximately 70–80$\%$ of most freshwater composition [57], and ATZ as a model pesticide. Accordingly, we determined the average R$_{\text {m}}$ of ATZ and HAs (in $\upmu$g$\cdot$d$^{-1}$), in the sorbent packed between membranes of different porosities (0,1, 5, and 8 $\mu$m), at identical conditions of target concentration of ATZ ($C_{\text {ATZ}}=20~\upmu \textrm{g} \cdot$L$^{-1}$), HA ($C_{\text {HA}}=4~\text {mg} \cdot$L$^{-1}$), water velocity ($V=0.3$ m$\cdot$s$^{-1}$), and total deployment time ($t=7$, 14, and 21 days) as presented in Table 2.

**Table 2:** Comparison of measured mass accumulation rates, *R*_m_, of a model target analyte, ATZ, and a model interference, HAs, on OasisHLB packed inside POCISs equipped with PES membranes of different porosities and at different deployment durations

		Accumulation rates			Normalized rates to $\Phi$=0.1 $\upmu$m$^{\textit{e}}$		
$$t^{\textit{a}}$$	$$\Phi $$ $$^{\textit{b}}$$	$$R_{\text {m},\text {ATZ}}$$$$^{\textit{c}}$$	$$R_{\text {m},\text {HA}}$$$$^{\textit{c}}$$	$$\frac{\text {R}_{\text {m},\text {ATZ}}^{\textit{d}}}{\text {R}_{\text {m},\text {HA}}}$$	*A*=$$\frac{\text {R}_{\text {m},\text {ATZ}}(\Phi )}{\text {R}_{\text {m},\text {ATZ}} (0.1\mu \text {m})}$$	*H*=$$\frac{\text {R}_{\text {m},\text {HA}}(\Phi )}{\text {R}_{\text {m},\text {HA}} (0.1\mu \text {m})}$$	*A*/*H*
7	0.1	1.0±0.0	2.8±0.2	0.4±0.0	1.0±0.0	1.0±0.1	1.0±0.1
	5	2.8±0.1	4.8±0.2	0.6±0.0	2.8±0.1	1.7±0.1	1.6±0.1
	8	3.9±0.1	3.7±0.2	**1.1±0.1**	3.9±0.1	1.3±0.1	**3.0±0.3**
14	0.1	1.4±0.0	1.3±0.2	1.1±0.2	1.0±0.0	1.0±0.2	1.0±0.2
	5	4.4±0.0	2.7±0.1	1.6±0.1	3.1±0.0	2.1±0.3	1.5±0.2
	8	5.7±0.1	2.6±0.1	**2.2±0.1**	4.1±0.1	2.0±0.3	**2.0±0.3**
21	0.1	1.9±0.0	1.0±0.2	1.9±0.4	1.0±0.0	1.0±0.3	1.0±0.3
	5	4.8±0.2	1.7±0.1	2.8±0.2	2.5±0.1	1.7±0.4	1.5±0.3
	8	5.8±0.1	1.5±0.2	**3.9±0.3**	3.1±0.1	1.5±0.3	**2.0±0.4**

$^{a}$ *t* = deployment time given in days

$^{b}$ $\Phi$ = porosity of PES membrane given in $\upmu$m

$^{c}$Mass accumulation rates, $R_{\text {m}}$, given in $\upmu$g$\cdot$d$^{-1}$ with uncertainties representing standard deviation of triplicate measurements

$^{d}$Given in $\upmu$g[ATZ]$\cdot$ $\upmu$g[HA]$^{-1}$ and expressed with uncertainties propagated from the corresponding standard deviation of each term

$^{e}$Normalized rates are dimensionless and expressed with uncertainties propagated from the corresponding standard deviation of each term

Entries in bold are for highlighting purposes of the significant gain in accumulation rates of ATZ, compared to HA, on membranes with 8 $\upmu$m pore size

At each deployment duration, $R_{\text {m},\text {ATZ}}$ and $R_{\text {m},\text {HA}}$ both experienced an increase with pore size enlargement (see $R_{\text {m},\text {ATZ}}$ and $R_{\text {m},\text {HA}}$ in Table 2), affirming our prediction of an increased accumulation of NOM on the sorbent at larger pore sizes, as well as confirming our earlier findings for ATZ at 120 $\upmu$g$\cdot$L$^{-1}$. Yet, the ratio of both terms, i.e., $R_{\text {m},\text {ATZ}}$/$R_{\text {m},\text {HA}}$, indicates a more pronounced increase for ATZ sampling compared to HAs, in particular at longer deployment duration that amounted upto $R_{\text {m},\text {ATZ}}$/$R_{\text {m},\text {HA}} = 3.9\pm 0.3~\upmu$g[ATZ]$\cdot \upmu$g[HA]$^{-1}$. These results imply a higher selectivity of POCIS towards ATZ at higher pore sizes. This is demonstrated through the normalized accumulation rates of ATZ and HAs to the corresponding values at 0.1 $\upmu$m (*A*/*H* = from 2.0±0.4 to 3.0±0.3). These results are in agreement with observations of reduced background interferences in the GC-IRMS chromatograms (data not shown).

Size exclusion is conceivably the fundamental mechanism that explains these findings, where the large membrane pores enable small molecules like ATZ to pass through easily while retaining larger molecules such as HAs. For instance, Yuan and Zydney [58] reported that 60% of HA components have a molecular weight greater than 50 kDa, with the highest fraction around 300 kDa, constituting about 33% of the total available DOM. This vast molecular weight distribution significantly influences the diffusion of HAs through the PES membrane and slows its movement through the pores. Another factor influencing R$_{\text {m},\text {HA}}$, in both membrane and sorbent, is their interactions with the PES membrane surface. The high content of oxygen-containing functional groups in HAs, such as carboxylic, phenolic, and alcoholic -OH groups, may undergo electrostatic interaction with the negatively charged surface of the PES membrane, causing repulsion and increasing the HA rejection [59–62]. This viewpoint is further corroborated through the higher accumulation of HA on the PES membrane compared to ATZ (see $C_{\text {PES}}$/$C_{\text {HLB}}$ in Table S4). Nonetheless, the latter may foster a favorable environment for bacterial growth on the membrane, where a susceptibility to biofouling might lead to the transformation of the analytes of interest and, hence, a potential compromise of their isotopic signatures. It was, therefore, of utmost importance to investigate the risk of biofouling to assess its potential impact on the accumulation system.

### No significant impact of (bio)fouling on POCIS with 8 $\upmu$m pore size

The impact of biofouling in POCIS on mass accumulation rates remains unclear, as it was reported to cause a significant increase (approx. 55%) for alkylphenols [63], contrasting with a decrease [64, 65] or a negligible effect on hydrophilic compounds [64, 66–68]. Moreover, biofouling formation under environmental conditions could harbor specific bacteria known to degrade the target analyte and compromise its isotopic signature [54]. We investigated, therefore, the magnitude of biofouling of POCIS with PES membranes of 0.1, 5, and 8 $\mu$m pore sizes through SEM images of the membrane surface and analysis of dry mass increase. Further, we investigated the influence of biofouled membranes on $R_{\text {m}}$ and the carbon isotope integrity of ATZ ($C_{\text {ATZ}}=2$ $\upmu$g$\cdot$L$^{-1}$) at a constant water velocity (*V*= 0.3 m$\cdot$s$^{-1}$), and a deployment time of 9 days. To this end, POCISs were initially prefouled in river water for 21 days and were then deployed in deionized water spiked with ATZ and stirred for another 9 days. For comparison, a control experiment was conducted without pre-fouling the POCISs.

**Fig. 4:**
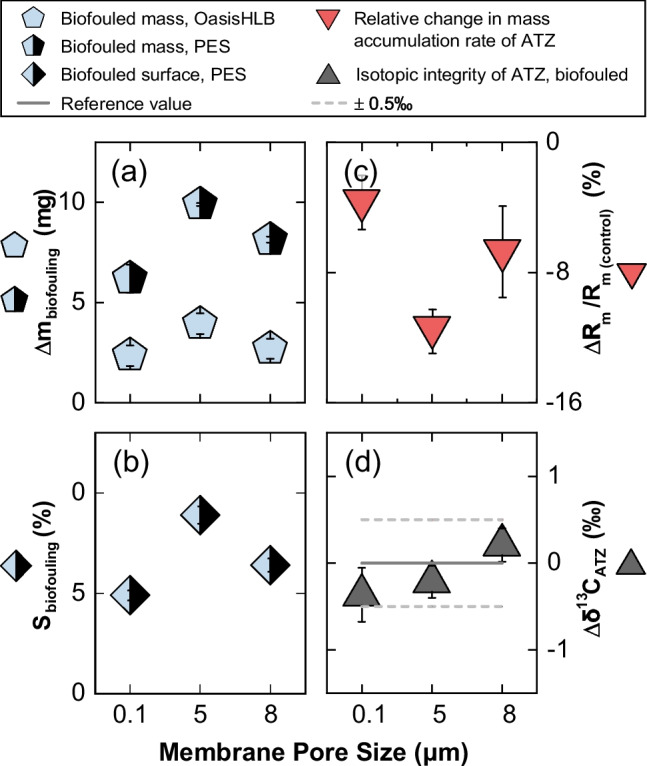
Assessment of the impact of membrane pore size on POCIS biofouling through **a** the increase in dry mass, $\Delta$m$_{\text {biofouling}}$, on the PES membrane (shaded symbols) and the Oasis HLB sorbent (open symbols), **b** the covered surface area of the biofouled PES membrane, S$_{\text {biofouing}}$, **c** the relative change in mass accumulation rates of ATZ on the Oasis HLB sorbent, $\Delta$R$_{\text {m}}=$R$_{\text {m}(\text {control})}-$R$_{\text {m}(\text {biofouled})}$, for the biofouled and non-fouled control, and **d** carbon isotope integrity of ATZ extracted from the sorbent of biofouled POCIS

The dry mass accumulated on the PES and OasisHLB during deployment was used as a proxy for biofouling ($\Delta$m$_{\text {biofouling}}$). A more significant increase in dry mass was observed on the membrane compared to the sorbent (Fig. 4a), consistent with the PES membrane’s observed rejection of HA species. SEM images of PES membrane surfaces corroborate these findings, revealing foreign deposits such as colloids and biomass (Figure S1). Quantification of these deposits via image analysis software showed surface coverage ($S_{\text {biofouling}}$) patterns that aligned with the dry mass increase (Fig. 4b). These results suggest a slight increase in biofouling susceptibility for larger pore sizes, particularly at 5 $\upmu$m. Mass accumulation rates of ATZ experienced a slight decrease upon fouling that amounted to 12±1% for 5 $\upmu$m. In contrast, a smaller decrease of 7±3% was observed for 8 $\upmu$m compared with 4±2% for 0.1 $\upmu$m ($\Delta$ $R_{\text {m}}$/$R_{\text {m} (\text {control})}$, Fig. 4c). Nonetheless, no isotopic fractionation was observed for carbon measurements of ATZ ($\Delta \delta ^{13}$C$_{\text {ATZ}} = -0.4\pm 0.3$ to $+0.2\pm 0.2$‰) as shown in Fig. 4d. Note that the accumulated mass of ATZ on the sorbent was insufficient for nitrogen isotope measurements. Mass balance calculations confirm, however, that no losses occurred during the entire experiment upon comparing the control (102.8±1.4%) with the biofouled experiment (98.5±1.6%), dismissing the possibility of any significant (bio)transformation of ATZ occurring at this level of biofouling. The Rayleigh equation further supports these results, considering the strongest isotope effects reported for ATZ biotransformation ($\epsilon _{\text {C}} = -5.5$‰ [17] and $\epsilon _{\text {N}} = +3.3$‰ [69]). Indeed, at least 9% and 29% of ATZ would have to transform in order to observe an isotopic shift on carbon and nitrogen beyond the typically accepted uncertainties, respectively. Although no significant biofouling was observed in the current work, further studies are warranted to account for the potential impact of varying environmental conditions, such as water chemistry and microbial activity, on the sampler’s performance.

## Conclusion

This study explores the role of membrane porosity in the uptake of selected pesticides for isotope analysis. The significant enhancement in mass accumulation rates at larger membrane porosities, along with the unchanged integrity of C and N isotope signatures, offers CSIA higher sensitivity and is conceivably extendable to other compounds. The affinity of the studied compounds towards the PES membrane seems to play a vital role in governing their mass accumulation rates on the POCIS and, hence, the observed enhancement. Here, we speculate that the balance between the affinity towards the membrane, compared to the one towards the sorbent, is the main driver of this enhancement. Furthermore, we conclude that passive sampling at larger pore sizes offers CSIA more selectivity, as evidenced by the relative mass accumulation rates of ATZ and HAs at the different pore sizes. Although no significant evidence on the impact of larger pore size (8 $\upmu$m compared with 0.1 $\upmu$m) on biofouling susceptibility has been found, this could vary depending on the water chemistry, matrix type, and target analyte and requires thus careful assessment on a case-by-case basis. Also, future studies should address the possible uptake of analyte fractions bound to particles or sediments when using larger pore size membranes (>0.1 $\upmu$m PES) to ensure accurate analyte representation, as well as the impact of flow-dependent sampling rates on the isotopic integrity of the sampled analytes. Further enhancement of mass accumulation rates can be explored through an increase in the sorbent mass, which could increase sensitivity by an additional factor of 1.6 (Figure S2 and S3). The higher sensitivity and selectivity offered by this approach is a step closer to the implementation of CSIA for aquatic contaminants occurring at low concentrations in the field.

## Supplementary Information

Below is the link to the electronic supplementary material.

Supplementary file 1 (pdf 1209 KB)
