# Supplementary material for: Role of membrane porosity in passive sampling of aquatic contaminants for stable isotope analysis: enhancement of analyte accumulation rates and selectivity
Source: Anal Bioanal Chem. 2025 Jan 31;417(8):1663–75. doi: 10.1007/s00216-025-05756-9 (PMC11876256; doi:10.1007/s00216-025-05756-9)
Supplement: Supplementary file 1 — (pdf 1209 KB) [file 216_2025_5756_MOESM1_ESM.pdf]

Supporting Information for

Analytical and Bioanalytical Chemistry

Role of Membrane Porosity in Passive Sampling of Aquatic Contaminants for Stable Isotope Analysis: Enhancement of Analyte Accumulation Rate and Selectivity

Armela Tafa[1], Anat Bernstein[2], Martin Elsner[1], and Rani Bakkour[1*]

[1]TUM School of Natural Sciences, Chair of Analytical Chemistry and Water Chemistry, Technical University of Munich, 85748 Garching, Germany.
[2]The Zuckerberg Institute for Water Research, The Jacob Blaustein Institutes for Desert Research, Ben-Gurion University of the Negev, Sde Boker Campus, 84990, Israel

*Corresponding author: rani.bakkour@tum.de
phone +49 89 289 54502, fax +49 89 2180 78255

9 Pages, 3 Figures, 4 Tables

Contents

S1 Chemicals, Materials, and Standard Solutions 3

S2 GC Temperature Program and MS Acquisition of Pesticides 5

S3 Expanded List of Potential Compounds Relevant to This Study 6

S4 $C_{PES}/C_{Sorbent}$ of HA and ATZ 7

S5 SEM images of (Bio)Fouled PES Membranes 7

S6 Impact of the Sorbent Mass on Accumulation Rates of ATZ 7

S1 Chemicals, Materials, and Standard Solutions

Table S1 provides information about chemicals, reagents, analytical standards, and sorbents used in this work. Stock solutions of analytical and isotopically labeled standards were prepared in methanol at a concentration of 1 g.L^{-1} and stored in darkness at -20 °C for further use. Standard solutions of humic acids (HA) were prepared by dissolution of 1 g of the powder in 1 L of MilliQ water at a pH of 10 while stirring for 3 hours, followed by filtering through 0.45 µm nylon membrane filters (47 mm, GVS, USA). After adjustment of the resulting filtrate to a pH value of 7, concentration of humic acid was confirmed using a TOC analyser and was stored in darkness at 4°C. Milli-Q water purification system (Merck Millipore, USA) was used for production of ultrapure water.

Table S1 List of chemicals and materials used in this work

Chemical	Specifications	Supplier
Reagents		
potassium hydrogen phthalate	≥99.95% & Sigma-Aldrich, Germany	
sodium hydroxide	≥98%	Sigma-Aldrich, Germany
hydrochloric acid	≥98%	Sigma-Aldrich, Germany
humic acid	≥99.5%	Sigma Aldrich, Germany
Solvents		
methyl tertiary-butyl ether	≥99.8%	Sigma-Aldrich, Germany
dichloromethane	EMSURE®, for analysis	Sigma-Aldrich, Germany
methanol	≥99%	Sigma-Aldrich, Germany
Analytical Standards		
atrazine (ATZ)	PESTANAL®	Sigma-Aldrich, Germany
terbutylazine	PESTANAL®	Sigma-Aldrich, Germany
boscalid (BOS)	PESTANAL®	Sigma-Aldrich, Germany
boscalid-d4	PESTANAL®	Sigma-Aldrich, Germany
S-metolachlor (MET)	PESTANAL®	Sigma-Aldrich, Germany
Sorbent & Membranes		
OasisHLB (N-vinylpyrrolidone-divinylbenzene)	particle size 30 µm. & pore size = 80Å	Waters
PES membrane filter Φ^a = 0.1 µm	D^b = 90 mm, t^c = 110–150 µm	Sterlitech Corporation, USA
PES membrane filter Φ^a = 5 µm	D^b = 90 mm, t^c = 110–150 µm	Sterlitech Corporation, USA
PES membrane filter Φ^a = 8 µm	D^b = 90 mm, t^c = 110–150 µm	Sterlitech Corporation, USA
polypropylene SPE cartridges	V^d = 6 mL	Altmann Analytik, Germany
polyethylene SPE frits	Φ^a = 20 µm	Altmann Analytik, Germany

[a] Φ = porosity;
[b] D = diameter;
[c] t = thickness;
[d] V = volume.

S2 GC Temperature Program and MS Acquisition of Pesticides

Temperature program for separation of ATZ, MET, and BOS on GC-MS was: 90 °C (0–1 min); 90–250 °C (1–9 min); 250 °C (9–12 min); 250–300 °C (12–17 min); 300 °C (17–20 min). Information on calibration (range = 0.03–1.2 mg.L^{-1}) are provided for each pesticide in Table S2, along with LODs and LOQs in the measured extracts determined after the calibration method DIN 32645[1].

Table S2 Limits of detection and quantification, quantifier ions, and regression calibrations for the investigated analytes after extraction and measurement on GC-MS

STD*	Quantifier (m/z)	RT* (min)	ISTD*	Calibration regression* (R^2)	LOD	LOQ
ATZ	200	7.77	TBZ	$y = 0.0017x - 0.1176$ (0.9992)	0.05	0.17
MET	162	9.03	TBZ	$y = 0.0085x - 1.5263$ (0.9893)	0.04	0.16
BOS	140	13.73	BOS-d4	$y = 0.0084x + 8.8325$ (0.9952)	0.04	0.15

* STD = standard; ATZ = atrazine; MET = S-metholachlor; BOS = boscalid; RT = retention time; ISTD = internal standard; TBZ = terbuthylazine; y = obtained ratio of peak areas of STD and ISTD on MS; x = concentration of standard; LOD and LOQ = limit of detection and quantification, respectively, given in mg·L^{-1}.

S3 Expanded List of Potential Compounds Relevant to This Study

Table S3 Selection of compounds expected to experience an increase in their mass accumulation rates on POCIS sampler packed with OasisHLB sorbent between two PES membranes of 8 μm posrosity.

Compound	log K_{ow}	E	S	A	B	V	log $K_{HLB,Water}$	log $K_{PES,Water}$
alachlor	2.95	1.16	1.20	0.00	1.15	2.1402	4.89[2]; 5.58±0.94[b]	3.33[2]; 3.44±0.64[c]
**atrazine**[a]	2.65	1.22	1.29	0.17	1.01	1.6196	4.15±0.82[b]; 4.68[2]; 4.79±0.06[3]	3.25[4]; 3.31[5]; 3.32±0.55[c]
**boscalid**[a]	2.96	n.a.[d]	n.a.[d]	n.a.[d]	n.a.[d]	n.a.[d]	5.31±0.04[3]	n.a.[d]
carbamazepine	2.45	2.15	1.90	0.50	1.15	1.8106	4.5[2]; 5.51±1.05[b]; 5.64[6]	2.47[4]; 2.86[2]; 3.45±0.68[c]
carbendazim	1.48	2.00	1.45	0.45	1.00	1.3613	4.34±0.88[b]; 4.87[6]	3.33[4]; 3.58±0.58[c]
cyanazine	2.22	1.41	2.00	0.22	1.14	1.7743	4.36±0.98; 5.36[6]; 4.51[2]	2.83[2]; 3.07±0.64[c]
cyproconazole	2.90	1.93	1.60	0.32	1.40	2.1618	5.93±1.08	3.04[2,5]; 3.15±0.73[c]
furalaxyl	2.61	1.49	1.45	0.00	1.60	2.3203	4.66[2]; 5.30[6]; 5.51±1.08[b]	2.81±0.76[c]; 2.97[6]
simazine	2.18	1.25	1.32	0.18	0.98	1.4787	3.79±0.80[b]; 4.45[2]; 5.35[6]	3.28[5]; 3.33±0.54[c]; 3.61[2,6]
**S-metolachlor**	3.13	1.15	0.95	0.09	1.35	2.28	5.01±0.02[3];5.57±0.97[b]	2.7[4]; 2.99±0.68[c] 3.05[5]
terbuthylazine	3.21	1.19	1.26	0.14	0.91	1.7605	4.81±0.06[3]; 4.85±0.84[b]	3.24[4]; 3.62±0.55[c]
terbutryn	3.74	1.43	1.23	0.12	0.99	1.9425	4.5[2]; 5.39[6]; 5.67±0.90[b]	3.39[2]; 3.48[4]; 3.80±0.60[c]

[a] compounds investigated in this study;
[b] estimated according to Dias and Poole 2002[7], where log $K_{HLB,Water}$ = (1.62±0.23)E − (0.36±0.28)S −(0.66±0.24)A − (2.47±0.27)B + (3.32±0.33)V − (0.13±0.33);
[c] estimated according to Suchana and Passeport 2022[8], where log $K_{PES,Water}$ = (1.00±0.14)E − (0.22±0.19)S −(1.35±0.30)A − (2.20±0.20)B + (0.47±0.22)V + (4.07±0.36);
[d] n.a. = not available.

S4 $C_{PES}/C_{Sorbent}$ of HA and ATZ

Table S4 Ratios of the concentration in the PES membrane over OasisHLB sorbent using the notion $C_{PES}/C_{OasisHLB}$ at different deployment duration.

Analyte	Porosity	7d	14d	21d
	0.1	0.7±0.1	1.0±0.2	0.8±0.2
HA	5	0.6±0.0	0.8±0.0	0.8±0.1
	8	0.7±0.1	0.7±0.0	0.7±0.1
	0.1	0.2±0.0	0.3±0.0	0.3±0.0
ATZ	5	0.1±0.0	0.1±0.0	0.1±0.0
	8	0.0±0.0	0.1±0.0	0.1±0.0

S5 SEM images of (Bio)Fouled PES Membranes

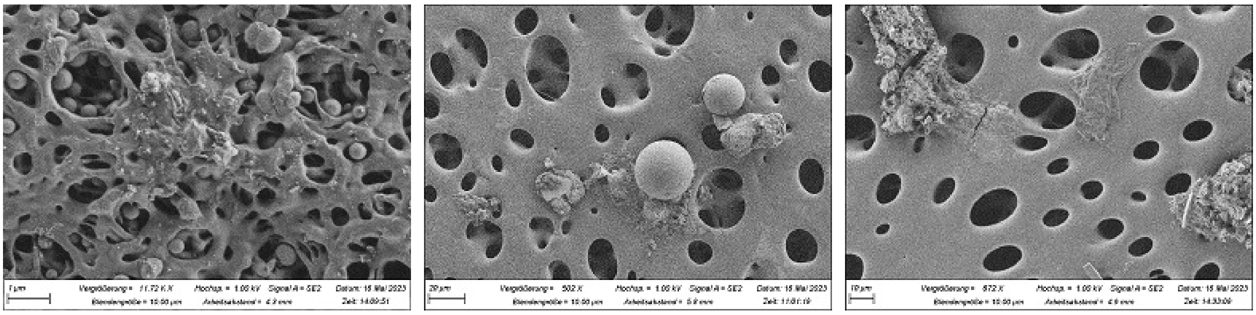

Figure S1 SEM images for biofouled PES membranes of different porosities of 0.1 (left), 5 (middle) and 8 µm (right).

S6 Impact of the Sorbent Mass on Accumulation Rates of ATZ

In this experiment, we increased the sorbent mass from 200 to 600, and 1200 mg and measured the mass accumulation rates of ATZ at two initial concentrations (C_{ATZ}=1 and 10 µg.L^{-1}) on POCIS equipped with PES membrane of 8µm porosity. We observed a consistent 1.6-fold increase in mass accumulation rate as the mass increased from 200 to 600 mg (Figure S3) –a phenomenon previously explained by Fauvelle et al.[9], while no increase occurred with further increase in the sorbent mass.

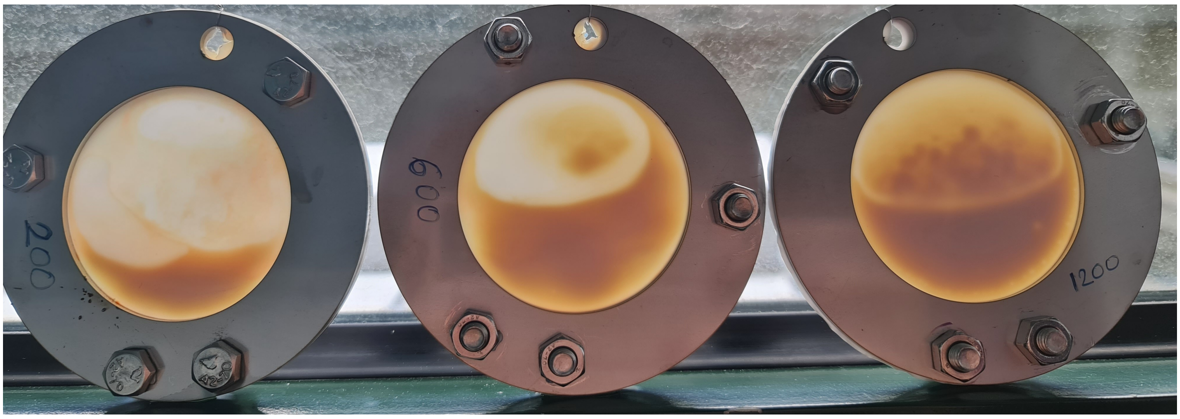

Figure S2 Employed POCIS (PES=8µm) with different sorbent masses of 200 (left), 600 (middle), and 1200 (right) with a distinctive pattern where the sorbent primarily accumulates in the lower section of the passive sampler.

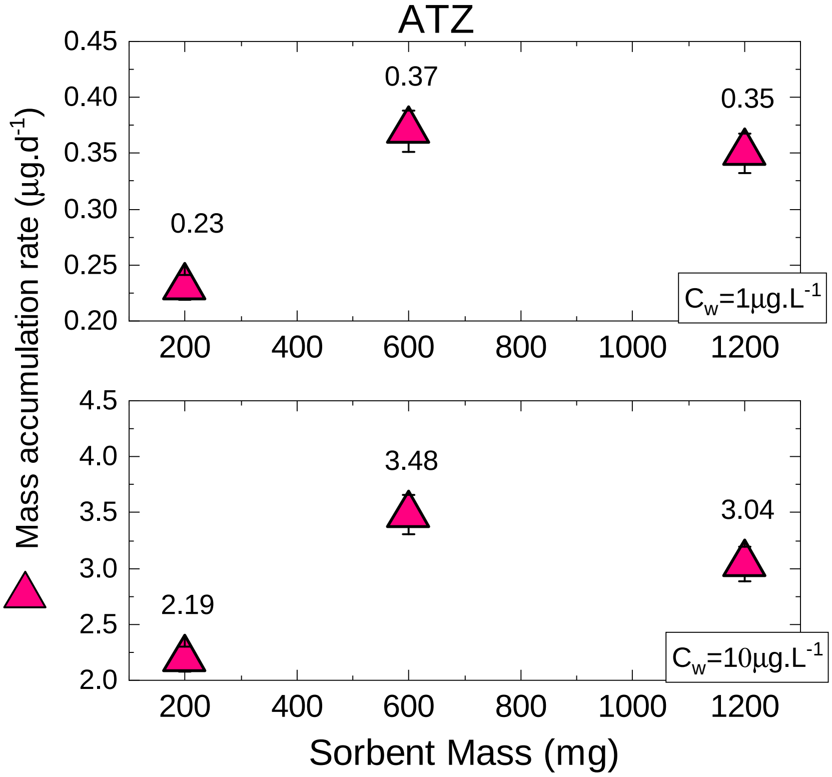

Figure S3 Atrazine mass accumulation rate of POCIS (PES=8µm) recorded in two different concentration setups (1 and 10μg/L) with increasing the sorbent mass between 200, 600 and 1200.

References

[1] DIN 32645: Chemical analysis – Decision limit, detection limit and determination limit under repeatability conditions – Terms, methods, evaluation. 2008.

[2] Jeong, Y.; Schäffer, A.; Smith, K. Comparison of the sampling rates and partitioning behaviour of polar and non-polar contaminants in the polar organic chemical integrative sam-

pler and a monophasic mixed polymer sampler for application as an equilibrium passive sampler. *Science of The Total Environment* **2018**, *627*, 905–915.

[3] Glöckler, D.; Wabnitz, C.; Elsner, M.; Bakkour, R. Avoiding Interferences in Advance: Cyclodextrin Polymers to Enhance Selectivity in Extraction of Organic Micropollutants for Carbon Isotope Analysis. *Analytical Chemistry* **2023**, *95*, 7839–7848.

[4] Vermeirssen, E. L. M.; Dietschweiler, C.; Escher, B. I.; van der Voet, J.; Hollender, J. Transfer Kinetics of Polar Organic Compounds over Polyethersulfone Membranes in the Passive Samplers Pocis and Chemcatcher. *Environmental Science & Technology* **2012**, *46*, 6759–6766.

[5] Djomte, V. T.; Chen, S.; Chambliss, C. K. Effects of suspended sediment on POCIS sampling rates. *Chemosphere* **2020**, *241*, 124972.

[6] Jeong, Y.; Schäffer, A.; Smith, K. Equilibrium partitioning of organic compounds to OASIS HLB® as a function of compound concentration, pH, temperature and salinity. *Chemosphere* **2017**, *174*, 297–305.

[7] Dias, N. C.; Poole, C. F. Mechanistic study of the sorption properties of OASIS® HLB and its use in solid-phase extraction. *Chromatographia* **2002**, *56*, 269–275.

[8] Suchana, S.; Passeport, E. Implications of polar organic chemical integrative sampler for high membrane sorption and suitability of polyethersulfone as a single-phase sampler. *Science of The Total Environment* **2022**, *850*, 157898.

[9] Fauvelle, V.; Mazzella, N.; Belles, A.; Moreira, A.; Allan, I. J.; Budzinski, H. Optimization of the polar organic chemical integrative sampler for the sampling of acidic and polar herbicides. *Analytical and Bioanalytical Chemistry* **2014**, *406*, 3191–3199.
